# Supplementary material for: The causal relationship between the human gut microbiota and pyogenic arthritis: a Mendelian randomization study
Source: Front Cell Infect Microbiol. 2024 Nov 26;14:1452480. doi: 10.3389/fcimb.2024.1452480 (PMC11629706; doi:10.3389/fcimb.2024.1452480)
Supplement: Supplementary file 1 [file DataSheet1.zip › mendelian test/ebi-a-GCST90027827.csv_forest.pdf]

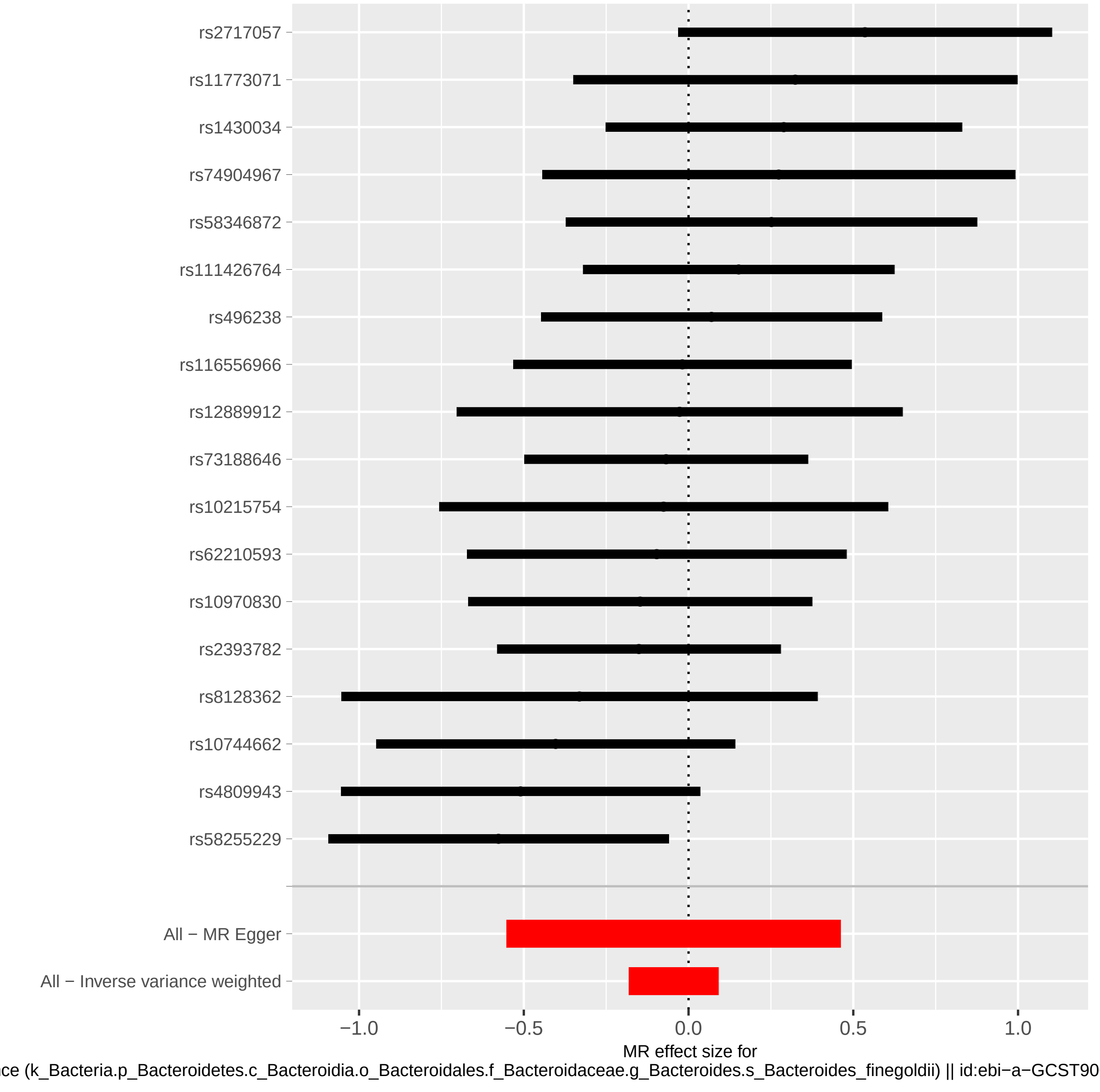
rs2717057
rs11773071
rs1430034
rs74904967
rs58346872
rs111426764
rs496238
rs116556966
rs12889912
rs73188646
rs10215754
rs62210593
rs10970830
rs2393782
rs8128362
rs10744662
rs4809943
rs58255229
All − MR Egger
All − Inverse variance weighted
−1.0
−0.5
0.0
0.5
1.0
MR effect size for
ce (k_Bacteria.p_Bacteroidetes.c_Bacteroidia.o_Bacteroidales.f_Bacteroidaceae.g_Bacteroides.s_Bacteroides_finegoldii) || id:ebi−a−GCST90
